# First Report of a SARS-CoV-2 Genome Sequence with a Spike His69-Val70 Deletion and an Asn439Lys Mutation in Morocco

**DOI:** 10.1128/MRA.00027-21

**Published:** 2021-03-18

**Authors:** Tarik Aanniz, Mouna Ouadghiri, Mohammed Amine Bendahou, Mohammed Walid Chemao-Elfihri, Mohamed Chenaoui, Hanae Dakka, Afaf Allaoui, Otmane Touzani, Amina Benaouda, Bouchra Belefquih, Saaïd Amzazi, Lahcen Belyamani, Azeddine Ibrahimi

**Affiliations:** a Medical Biotechnology Laboratory, Bioinova Research Center, Rabat Medical and Pharmacy School, Mohammed Vth University, Rabat, Morocco; b Research Center of Plant and Microbial Biotechnologies, Biodiversity, and Environment, Faculty of Sciences, Mohammed Vth University, Rabat, Morocco; c Laboratoire d’Analyse Médicales Biolife, Harhoura, Temara, Morocco; d Laboratoire de Biologie des Pathologies Humaines, Faculté des Sciences, Mohammed Vth University, Rabat, Morocco; e Laboratoire de Biodiversité, Écologie, et Génome, Faculté des Sciences, Mohammed Vth University, Rabat, Morocco; f Laboratoire Touzani d'Analyses Biologiques et Médicales, Temara, Morocco; g Microbiology Laboratory, Cheikh-Zaid University Hospital, Abulcasis University of Health Sciences, Rabat, Morocco; h Laboratory of Human Pathologies Biology, Faculty of Sciences, Mohammed Vth University, Rabat, Morocco; i Emergency Department, Military Hospital Mohammed V, Rabat Medical and Pharmacy School, Mohammed Vth University, Rabat, Morocco; DOE Joint Genome Institute

## Abstract

We report the nearly complete genome sequence and the genetic variations of a clinical sample of severe acute respiratory syndrome coronavirus 2 (SARS-CoV-2) collected from a nasopharyngeal swab specimen from a male patient from Harhoura-Rabat, Morocco. The sequence, which was obtained using Ion Torrent technology, is valuable as it carries a recently described deletion (His69-Val70) and substitution (Asn439Lys).

## ANNOUNCEMENT

The pandemic of coronavirus disease 2019 (COVID-19) continues to spread worldwide. The use of genomic data in conjunction with epidemiological data can facilitate early decisions for the control of transmission of severe acute respiratory syndrome coronavirus 2 (SARS-CoV-2), belonging to the *Betacoronavirus* genus and *Coronaviridae* family (1–3). We used the Ion S5 next-generation sequencing (NGS) technology for whole-genome sequencing (WGS) to detect new mutants that are currently spreading and attracting interest in Europe, mainly in the United Kingdom, and in South Africa (4–6).

A clinical sample of SARS-CoV-2 was collected in December 2020 by BioLife Laboratory (Harhoura-Rabat, Morocco) from a nasopharyngeal swab specimen from a male patient from Harhoura-Rabat, Morocco. RNA extraction was performed using the MagaBio plus virus DNA/RNA purification kit II (BioFlux, China). The patient was initially identified as positive for COVID-19 by reverse transcriptase quantitative PCR and exhibited cycle threshold (*C_T_*) values of 22.31, 27.24, and 23.53 for the N, RdRp, and E genes, respectively. Then, the cDNA was prepared using the SuperScript VILO cDNA synthesis kit (Invitrogen, Thermo Fisher Scientific, USA). Fifteen microliters of cDNA was used to prepare a SARS-CoV-2 library by using an Ion AmpliSeq kit for Chef DL8 (Thermo Fisher Scientific). The library was adjusted to 30 pM and then loaded onto an Ion Chef instrument (Thermo Fisher Scientific) for emulsion PCR, enrichment, and loading onto an Ion 530 chip. WGS was performed using the Ion AmpliSeq SARS-CoV-2 research panel designed by Thermo Fisher Scientific for complete viral genome sequencing according to the instructions for use on an Ion GeneStudio S5 Prime Series system.

Raw data were analyzed using Torrent Suite software v5.12.0, and the NGS QC Toolkit v 2.3.3 was used to remove low-quality and short reads. Variant Caller v5.10.1.19 was used to detect variants, compared to the reference genome (Wuhan-Hu-1 strain [GenBank accession number MN908947.3]), and the consensus sequence was generated using IRMAreport v1.3.0.2. The annotation was performed using COVID19AnnotateSnpEff v1.3.0.2, a plugin specifically developed for SARS-CoV-2 that can predict the effect of a base substitution (7).

Our findings allowed us to obtain a SARS-CoV-2 genome of 29,826 bp from 1,392,344 reads; 1,373,947 reads were mapped, covering 98.42% of the total genome with a mean depth of 8,863×. The DNA G+C content was 37.99%. Genetic variant analysis revealed a total of 21 mutations, including 7 synonymous and 10 missense variants (Table 1). The spike harbored the disruptive in-frame deletion known as the His69-Val70 deletion. Moreover, an upstream open reading frame 1ab (ORF1ab) mutation at position 241, an upstream ORF8 mutation at position 27800, and a downstream S mutation at position 29734 were reported (Table 1).

**TABLE 1:** Types and effects of identified gene variations

Residue change	Nucleotide position	Nucleotide change	Variation type^*a*^	Gene	Effect
No change assigned	241	c.−25C>T	SNP	ORF1ab	Upstream gene variant
p.Leu88Leu	527	c.262C>T	SNP	ORF1ab	Synonymous variant
p.Phe924Phe	3037	c.2772C>T	SNP	ORF1ab	Synonymous variant
p.Ile2501Thr	7767	c.7502T>C	SNP	ORF1ab	Missense variant
p.Tyr2594Tyr	8047	c.7782C>T	SNP	ORF1ab	Synonymous variant
p.Leu3754Phe	11527	c.11262G>T	SNP	ORF1ab	Missense variant
p.Met4241Ile	12988	c.12723G>T	SNP	ORF1ab	Missense variant
p.Pro4715Leu	14408	c.14144C>T	SNP	ORF1ab	Missense variant
p.Val5112Ile	15598	c.15334G>A	SNP	ORF1ab	Missense variant
p.His5614Tyr	17104	c.16840C>T	SNP	ORF1ab	Missense variant
p.Ala5922Ser	18028	c.17764G>T	SNP	ORF1ab	Missense variant
p.Leu6668Leu	20268	c.20004A>G	SNP	ORF1ab	Synonymous variant
p.Asn6729Asn	20451	c.20187C>T	SNP	ORF1ab	Synonymous variant
p.His69_Val70del	21764	c.204_209 delACATGT	Deletion	S	Disruptive in-frame deletion
p.Asn439Lys	22879	c.1317C>A	SNP	S	Missense variant
p.Asp614Gly	23403	c.1841A>G	SNP	S	Missense variant
p.Thr1116Thr	24910	c.3348T>C	SNP	S	Synonymous variant
p.Ile1130Val	24950	c.3388A>G	SNP	S	Missense variant
p.Arg150Arg	26972	c.450T>C	SNP	M	Synonymous variant
No change assigned	27800	c.−94C>A	SNP	ORF8	Upstream gene variant
No change assigned	29734	c.*4350G>C	SNP	S	Downstream gene variant

a SNP, single-nucleotide polymorphism.

The His69-Val70 deletion (spike N-terminal domain) cooccurring with the Asn439Lys mutation (spike receptor binding domain) in the studied case was not reported in Morocco previously. The His69-Val70 deletion is one of the mutations reported for new emergent lineages primarily identified in the United Kingdom, while Asn439Lys was primally reported in Scotland and is now spreading worldwide. Many studies reported that both mutations enhanced binding affinity for the hACE2 receptor, increasing transmissibility, while showing similar clinical outcomes and *in vitro* replication fitness, compared to the wild-type strain (8–10).

### Data availability.

The consensus sequence generated by IRMAreport v1.3.0.2 was deposited in the GenBank and GISAID databases under the accession numbers MW453084 and EPI_ISL_728353, respectively. The raw reads were deposited in the NCBI Sequence Read Archive (SRA) under the accession number SRR13444960.
